# A Case of Short Stature and Severe Osteoporosis in a Young Man with Oculocutaneous Albinism: Syndrome or Coincidence?

**DOI:** 10.7759/cureus.7817

**Published:** 2020-04-24

**Authors:** Samson O Oyibo

**Affiliations:** 1 Internal Medicine, Peterborough City Hospital, Peterborough, GBR

**Keywords:** idiopathic short stature, osteoporosis, oculocutaneous hypopigmentation, albinism, growth hormone therapy, insulin-like growth factor 1, syndrome or coincidence, case report, low-trauma fractures, syndromic albinism

## Abstract

Oculocutaneous albinism (OCA) is a rare autosomal recessive congenital condition characterized by reduced or absent production of the pigment melanin by melanocytes. The affected individuals have increased susceptibility to sunburn and skin cancers. Osteoporosis is a disease entity characterized by the progressive loss of bone mineral density and the deterioration of bone micro-architecture, leading to an increased risk of developing low-trauma fractures. There are many causes of osteoporosis, ranging from primary to secondary causes. Short stature is defined as height less than two standard deviations below the age-specific and gender-specific mean (less than the 2.5th percentile). There have been rare case reports of individuals with OCA having associated osteoporosis or low bone mineral density and short stature. These cases have also been associated with severe skeletal, neurological, and psychomotor disabilities. This paper presents a case of a young man with OCA and short stature who sustained a low-trauma intertrochanteric fracture to his femur bone and was subsequently diagnosed to have clinically significant osteoporosis. This case report while attempting to review the literature also emphasizes the importance of further research into the prevalence of these clinical features accompanying certain types of OCA and whether they are part of a single syndrome or just coincidental findings.

## Introduction

Oculocutaneous albinism (OCA) is a rare autosomal recessive congenital condition characterized by reduced or absent production of the pigment melanin by melanocytes. The reduced or absent pigmentation affects the skin, hair follicle, and eyes (especially the iris). This results in photophobia, nystagmus, reduced visual acuity, and increased skin sensitivity to sunlight. There is also increased susceptibility to sunburn and skin cancers. As yet, there is no cure for OCA, but advice is given about skin and eye protection from direct sunlight [1].

Osteoporosis is a disease entity characterized by the progressive loss of bone mineral density (BMD) and the deterioration of bone micro-architecture, leading to an increased risk of developing low-trauma fractures. The causes of osteoporosis can be divided into primary (multifactorial, polygenic, and age-related) and secondary (drugs, metabolic diseases, endocrine disorders, smoking, malabsorption syndromes, prolonged immobility, bone disorder, etc.) [2]. There is a multitude of genetic diseases associated with osteoporosis, but clinical characterization is difficult because of the interaction between several environmental and genetic factors. The genes associated with the bone phenotype are distributed throughout the human genome and located in practically all chromosomes [3].

Short stature is defined as height less than two standard deviations (SDs) below the age-specific and gender-specific mean (less than the 2.5th percentile). There are several causes, of which endocrine disorders make up 5%. Growth hormone deficiency (GHD) or disorders of the growth hormone/insulin-like growth factor 1 (IGF-1) axis are rare endocrine causes in addition to thyroid failure and Cushing’s syndrome. The other causes include familial (short parents) short stature, delayed puberty, constitutional growth delay, small-for-gestational age, chronic illness, bony dysplasias, genetic conditions, and dysmorphic syndromes [4]. Full evaluation and genetic characterization according to guidelines are required for the accurate diagnosis and management of short stature in childhood [4].

The combination of OCA and osteoporosis as a separate condition has not been commonly reported. This paper presents a case of a young man with OCA and short stature who sustained a low-trauma intertrochanteric fracture to his femur bone and was subsequently discovered to have clinically significant osteoporosis.

## Case presentation

Medical history and demographics

**Figure 1:**
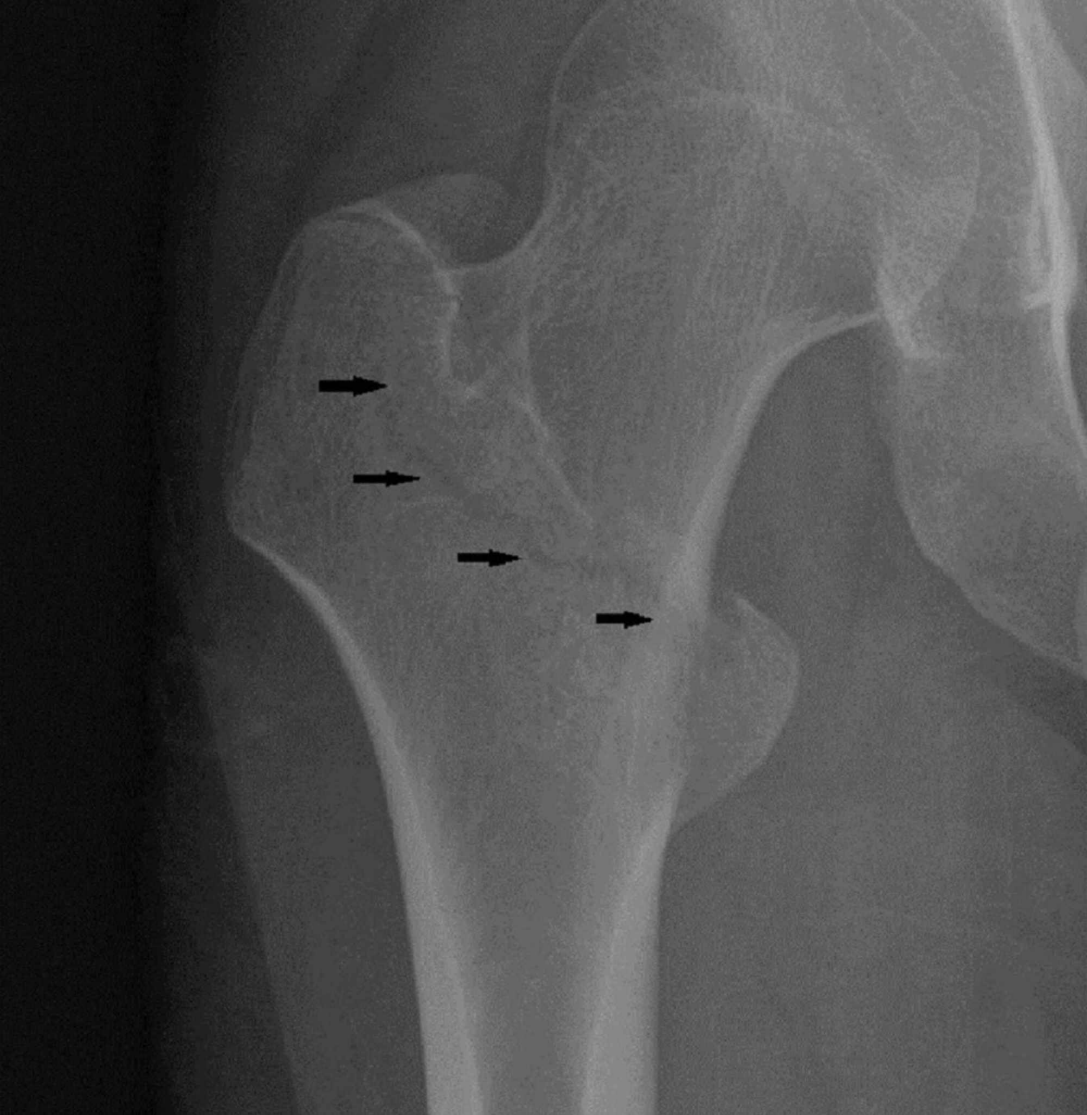
Radiograph showing an intertrochanteric non-displaced fracture (arrows) of the neck of the right femur bone prior to fixation

A 24-year-old man attended a routine diabetes clinic appointment and stated that he had sustained a low-trauma intertrochanteric fracture to his right femur bone during a fall from the lowest tread of a short flight of stairs (Figure 1). This had occurred a few months prior to his diabetes appointment. The fracture had been repaired using a dynamic hip screw procedure.

His medical history consisted of type 1 diabetes since the age of six years and congenital OCA (eye and skin hypopigmentation). He had no other chronic illness, no history of chronic corticosteroid usage during childhood, and no history of low-trauma fractures during childhood. His diabetes was well controlled on a basal-bolus insulin regimen (glargine and aspart). His parents were healthy, non-consanguineous, and of British origin. There was no family history of low-trauma fractures.

**Figure 2:**
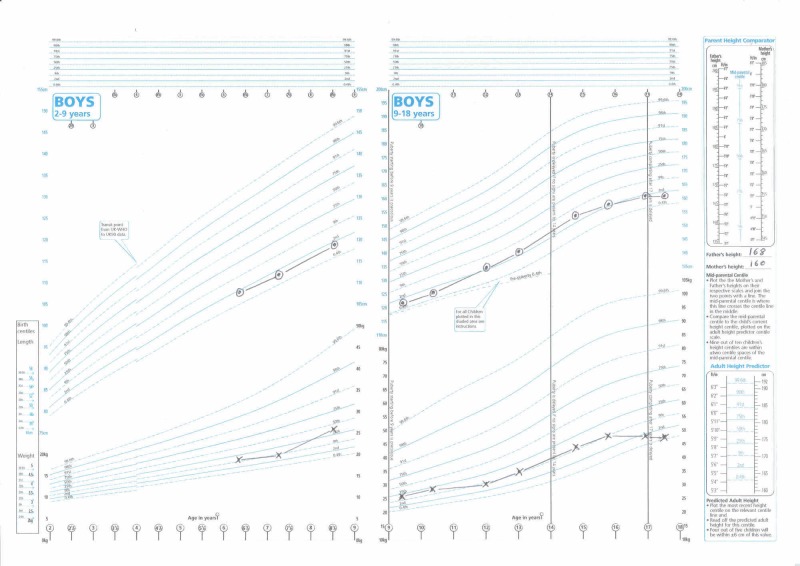
A growth chart showing the height (dotted circles) and weight (crosses) for the patient during 2-9 years of age and 9-18 years of age Source: UK-WHO growth charts (2-18 years) from the Royal College of Paediatrics and Child Health

He was born at 39 weeks’ gestation by normal vaginal delivery. His birth weight was 3.86 kg (84th percentile). He had a specialist review at the age of four months due to rapid growth in head circumference, demonstrating growth at two months to the 73rd percentile and further growth at four months to above the 97th percentile. A magnetic resonance imaging (MRI) scan performed at that time revealed no abnormalities or hydrocephalus. The patient’s father had a head circumference above the 97th percentile; therefore, this was put down to familial large head. At the age of eight months, he was noted to have delayed motor development (still requiring sitting support, no saving reaction, and not taking weight on his legs). At the age of 10 months, his body length was 71.3 cm (19th percentile) and weight was 9.26 kg (54th percentile). His height measurements charted over the years indicated a height growth below the 2.5th percentile from the age of six years onward (Figure 2). However, his height growth velocity was normal, with progression along a particular percentile line throughout childhood and adolescence.

Examination demonstrated a healthy young man with a weight of 51.5 kg and a height of 1.61 m (body mass index: 19.9 kg/m^2^). His height was below his expected final (mid-parental) height and below the 2.5th percentile. His expected mid-parental height (range) given his father’s height (1.68 m) and mother’s height (1.6 m) was 1.7 (1.62-1.79) m. He had short stature compared with his peers and siblings during childhood. He had pale skin, striking blonde hair, blonde eyebrows, and blonde eyelashes. He had pale iris and horizontal nystagmus. He had normal secondary sexual characteristics.

Investigations

The history prompted the performance of a BMD scan, which surprisingly revealed a severe low-for-age BMD (z-score = -3.6 SD and BMD = 0.695 g/cm^2^ in spine; z-score = -3.0 SD and BMD = 0.524 g/cm^2^ in femoral neck). Blood results revealed normal full blood count, serum calcium, phosphate, alkaline phosphatase, ferritin, magnesium, and 25-hydroxy vitamin D levels. The patient’s thyroid, liver, and renal function tests were also normal. An early morning serum cortisol assessment ruled out adrenal failure. Coeliac screen was negative, and his serum parathyroid hormone level was normal. His glycated hemoglobin level (52 mmol/mol) indicated adequate diabetes control. His serum prolactin was mildly elevated at 450 mU/L, but his serum testosterone and gonadotropin levels were all normal. His serum IGF-1 level was lower than the normal range for his age and gender (21- to 25-year-old males). The blood results are shown in Table 1. The patient’s 24-hour urinary assessment ruled out excess cortisol production or Cushing’s syndrome (Table 2).

**Table 1:** Results of routine hematological and biochemical investigations

Blood parameters	Normal range	Patient’s results
Hemoglobin (g/L)	130-180	149
White cell count (10^9^/L)	4.0-11.0	5.7
Platelets (10^9^/L)	150-400	255
Sodium (mmol/L)	135-145	139
Potassium (mmol/L)	3.4-5.1	3.8
Creatinine (µmol/L)	45-84	62
Adjusted calcium (mmol/L)	2.2-2.6	2.31
Phosphate (mmol/L)	0.8-1.5	1.05
Magnesium (mmol/L)	0.7-1.0	0.87
Alkaline phosphatase (U/L)	30-130	70
Parathyroid hormone (pmol/L)	1.4-6.2	2.3
25-hydroxy vitamin D (nmol/L)	>50	61
Thyroid-stimulating hormone (mU/L)	0.3-4.2	1.33
Free thyroxine (pmol/L)	12.0-22.0	15.7
Glycated hemoglobin (mmol/mol)	<48	52
Insulin-like growth factor (nmol/L)	24.4-52.0	17.5
Prolactin (mU/L)	<330	450
Follicle-stimulating hormone (U/L)	2-13	2
Luteinizing hormone (U/L)	2-9	8
Testosterone (nmol/L)	10.0-38.0	30.2
9 am cortisol (nmol/L)	250-600	345

**Table 2:** Result of a 24-hour urinary free cortisol excretion test

Urine parameter	Normal range	Patient’s result
24-hour urinary free cortisol excretion (nmol/L)	0-146	79

**Figure 3:**
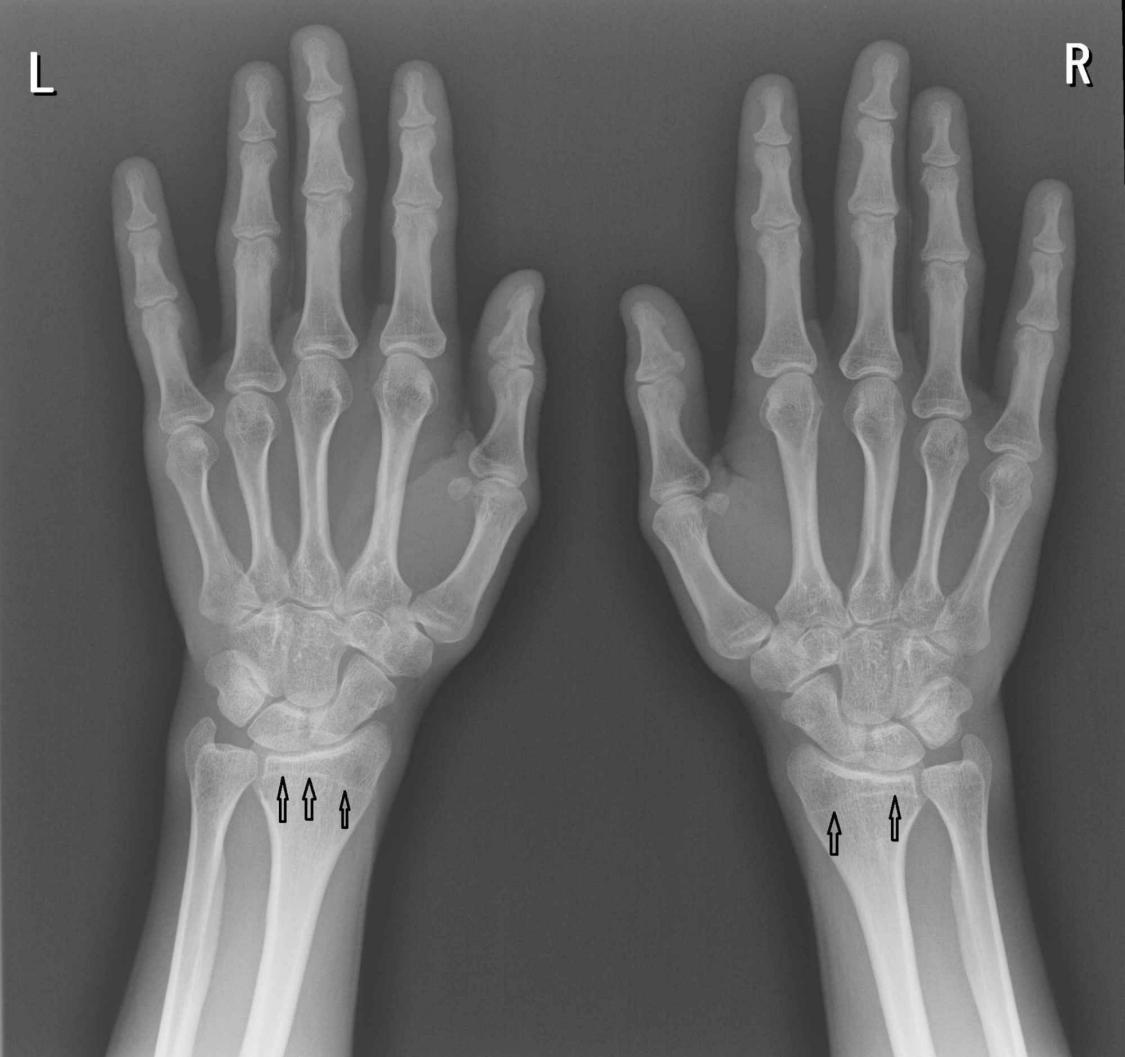
Radiograph of the left and right hands demonstrating epiphyseal closure plates (arrows) indicating full skeletal maturity

**Figure 4:**
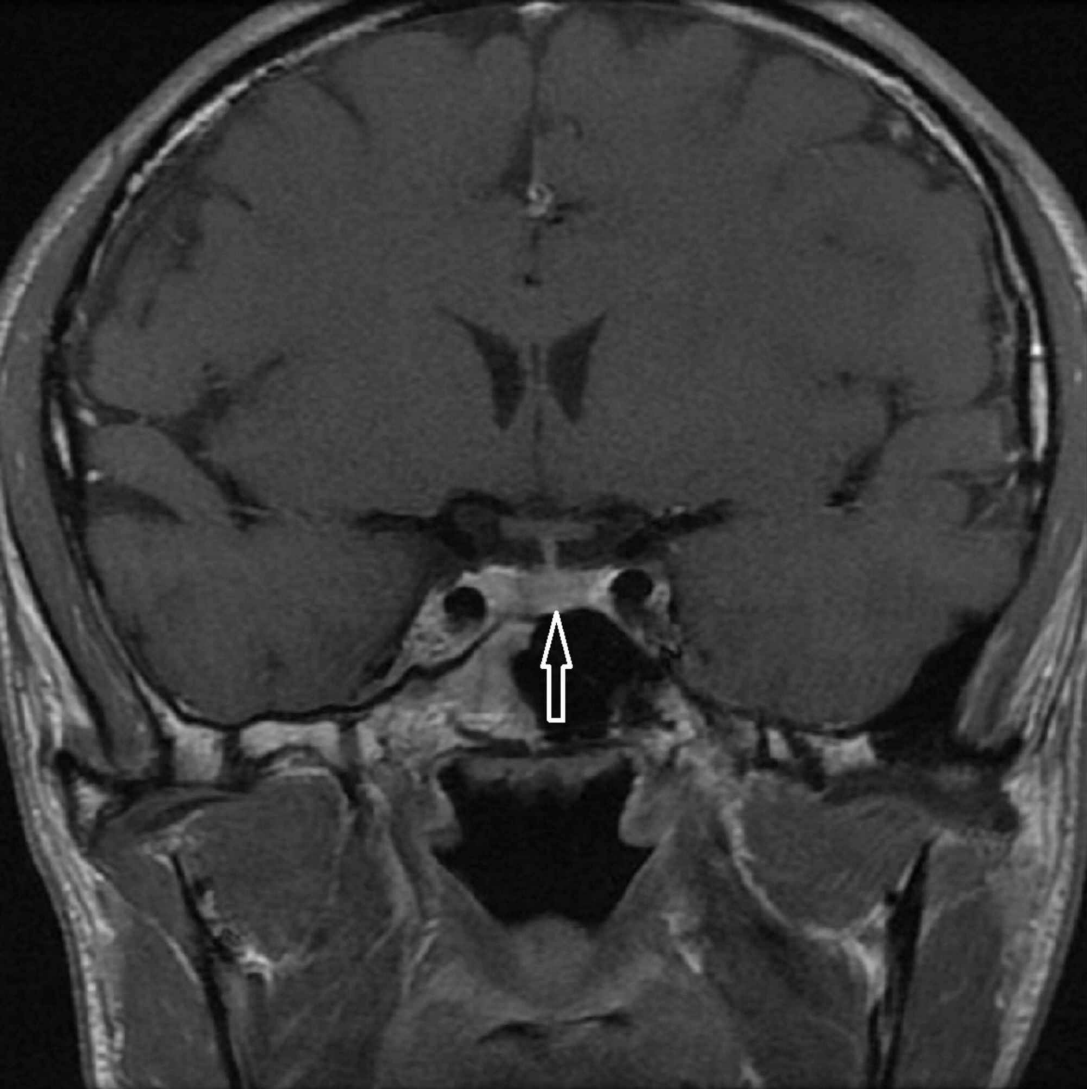
A magnetic resonance imaging (MRI) scan demonstrating a normal pituitary gland (arrow)

A radiograph (X-ray) of his hands revealed that all epiphyseal plates were closed consistent with full skeletal maturity (Figure 3). A contrast pituitary MRI scan demonstrated a normal pituitary gland size and configuration (Figure 4).

Treatment

The results were discussed with the patient, and he was initiated on an oral bisphosphonate (alendronic acid 70 mg weekly) and a calcium-vitamin D supplement Adcal-D3® (calcium 1,000 mg and vitamin D3 880 IU) one tablet daily. He was also advised to continue weight-bearing exercises.

Outcome and follow-up

His initial low-for-age serum IGF-1 level prompted a referral for formal growth hormone assessment and possible growth hormone therapy for the low BMD, but due to logistic reasons, the patient missed several appointments and was not keen to undergo further assessment. A repeat blood test performed nine months later revealed a normal serum prolactin level (182 mU/L) and a normal serum IGF-1 level of 18.8 nmol/L. Although the second serum IGF-1 level was only slightly higher than the previous result, it was now within the normal age- and gender-specific range (16.3-39.3 nmol/L) for 25- to 41-year-old males. A follow-up bone density scan was planned to assess the effect of treatment.

## Discussion

There are several types of albinism, which include ocular, oculocutaneous (OCA), and oculocerebral hypopigmentation syndromes, and other syndromic albinism, which have severe (some life-threatening) neurological and other organ affectation. However, OCA is the commonest type, affecting 1 in 17,000 people. There are four distinct types of OCA caused by distinct gene defects. These genes encode for tyrosinase (TYR), the melanosomal transmembrane protein (OCA2), tyrosinase-related protein-1 (TYRP1), and the membrane-associated transporter protein (MATP), which are all involved in melanin synthesis from the tyrosine precursor [1].

The combination of hypopigmentation and reduced bone density was first described in two siblings born to consanguineous Italian parents in 1983 [5]. These siblings also had severe skeletal deformities with severe growth and psychomotor retardation. A similar case with similar disabilities was also noted in an offspring of non-consanguineous British parents in 1987 [6]. The individuals in these case reports had more severe oculocerebral hypopigmentation as opposed to OCA.

The presence of osteoporosis in an individual with OCA was first described in an eight-year-old child born to non-consanguineous Mexican parents in 1996. The authors reported this as a new syndrome called osteoporosis-oculocutaneous hypopigmentation syndrome (OOCHS) or Hernàndez-Fragoso’s syndrome [7]. This child had short stature along with other skeletal (facial, spine, limbs, and chest) abnormalities. Another two cases of OOCHS were described in two siblings thereafter [8]. These were a brother and sister from healthy non-consanguineous parents of Balkan-Turkish origin. The male sibling had osteopenia, and the female sibling had osteoporosis. Both of them did not have short stature or any other abnormalities.

OOCHS is registered under the Orphanet portal for rare diseases (ORPHA: 2786, OMIM: 601220) with a prevalence of less than one in a million (<1/1,000,000) and the apparent mode of inheritance being autosomal recessive [9]. To date, only the above three cases have been described in the literature [7,8]. Therefore, this is the fourth reported case involving a patient who had a combination of osteoporosis, OCA, and short stature but without any accompanying severe skeletal or neurological abnormalities or psychomotor retardation. Additionally, the patient in this report had a low-for-age serum IGF-1 level that gave rise to a suspicion of possible GHD during childhood years.

The etiopathogenesis of the osteoporosis or low bone density in OCA has not been explained. This could be because of paucity of cases. In most of the reported cases, the osteoporosis was discovered during childhood [5-7]. From these reports, we cannot ascertain whether the osteoporosis was congenital or acquired during the childhood period. There is need for more research into the etiopathogenesis of osteoporosis or low bone density in individuals with OCA. Consequent to the paucity of cases and knowledge thereof, the treatment of the osteoporosis associated with OCA or its response to the standard treatments (bisphosphonates, vitamin D, and calcium) have not been evaluated.

Vitamin D is required for bone health and maintenance; therefore, vitamin D deficiency is a risk factor for osteoporosis. Ultraviolet B (UVB) radiation is absorbed by 7-dehydrocholesterol present in the plasma membrane of epidermal keratinocytes and dermal fibroblast, resulting in the formation of vitamin D3 [10]. It is known that melanin competes for the same UVB radiation such that individuals with increased skin melanin need more sun exposure to produce the same quantity of vitamin D3 compared with individuals with light skin [11]. Therefore, low melanin levels should not be a risk factor for osteoporosis. Additionally, the patient in this case report had normal 25-OH-vitamin D levels.

Our patient could have had other rare causes for osteoporosis. Idiopathic juvenile osteoporosis is characterized by pre-pubertal onset with recurrent long-bone fractures, back pain, and gait disturbances. It is usually self-limiting with spontaneous remission at the onset and progression of puberty [12]. The patient in this case report did not have a history of recurrent long-bone fractures in childhood.

Chronic diseases such as cystic fibrosis, thalassemia major, primary biliary cirrhosis, mastocytosis, mucopolysaccharidoses, Gaucher’s disease, galactosemia, and systemic sclerosis are other rare causes of osteoporosis [13]. However, the patient in this case report did not have any history or clinical features suggestive of these rare conditions.

Endocrine causes for osteoporosis, such as primary hyperparathyroidism, acromegaly, Cushing’s syndrome, hypogonadism, and hyperprolactinemia, were ruled out by normal endocrine tests [13]. It must be noted that the patient in this case report had a serum IGF-1 level below the age-specific and gender-specific reference range, generating a suspicion of isolated GHD, which is another rare endocrine cause of osteoporosis and short stature. GHD is present when there is a disruption of the growth hormone/IGF-1 axis due to a cause that can be acquired, congenital, or idiopathic. Congenital causes are generally due to genetic mutations. Acquired causes range from trauma, post-surgery, infections, hypothalamic and pituitary tumors, cranial irradiation, and other systemic diseases such as histiocytosis and tuberculosis. Growth-hormone-releasing hormone from the hypothalamus stimulates the pulsatile release of growth hormone from the anterior pituitary gland into the circulation. This, in turn, stimulates the release of IGF-1 from the liver and some target sites of growing tissue. Affected neonates can present with hypoglycemia, jaundice, and microcephalus. Older children present with short stature. Serum IGF-1 assessment is used to screen for GHD when the condition is suspected. However, provocative growth hormone testing in combination with clinical, auxological, radiological, and biochemical data is required when making a diagnosis [14]. It must be noted that IGF-1 is not always low in patients with GHD and that there are other causes of low serum IGF-1 levels, such as nutritional deficiency, chronic illness, and hypothyroidism. Low serum IGF-1 levels have also been reported in young people with diabetes, but the true association and mechanism remain unclear [15].

Studies have shown that GHD itself is crucial for the development of osteopenia in patients with hypopituitarism, and studies have shown the beneficial effects of growth hormone therapy on reduced BMD [16]. There are data suggesting that young adults with GHD, who have not reached lumbar peak bone mass at the time of discontinuation of growth hormone therapy, might present with low BMD if they are not treated during the transition period to young adulthood [17]. Recombinant growth hormone treatment has been demonstrated to improve BMD in men with idiopathic osteoporosis [18].

Short stature was noted in the cases of OCA reported above, where osteoporosis was discovered during childhood [5-7]. There was no mention of serum IGF-1 measurement or growth hormone assessment in these cases. However, in an earlier case report involving two siblings with OCA, dysmorphic features, and short stature, there is mention of a partial response to a growth hormone stimulation test in one of the siblings despite normal serum IGF-1 levels. The test could not be repeated and therefore growth hormone therapy was given to improve height [19]. The authors commented on the concomitant occurrence of short stature and albinism in two siblings, as well as consanguinity, raising the possibility of either a single autosomal recessive gene as the cause of the disorder or two contiguous genes.

There were no prior results to suggest low serum IGF-1 levels or GHD during childhood. However, historical height measurements do indicate evidence of short stature during the childhood to adolescent period. His mother’s height was on the 32nd percentile and father’s height on the 14th percentile; therefore, familial short stature is not a likely cause. He had progressive height growth along/parallel to a percentile range during childhood and adolescence, indicating normal growth velocity [20]. Despite this, he did not reach his genetically determined target height. Height growth measurement charts for the period between one and five years of age were not available; therefore, it was not possible to assess for any downward shift or reduction in growth velocity during infancy and early childhood. The initial serum IGF-1 level was lower than the age-specific and gender-specific reference range, but after a repeat test a year later, it was within the new age-specific and gender-specific reference range. In the absence of formal growth hormone/IGF-1 assessment during childhood/adolescence, one cannot confirm or dismiss the possibility of mild GHD occurring during early childhood.

The severe osteoporosis discovered in this patient was a coincidental finding, when a bone density scan was performed to assess bone health after the patient reported sustaining a low-trauma fracture of his femur bone. This case is dissimilar to previous cases in that the osteoporosis was detected during adulthood and this patient had a short stature. The osteoporosis may have accompanied the short stature over several years during childhood and adolescence and could be due to a primary or congenital cause related or unrelated to OCA. We cannot predict what the response will be to the standard treatment for osteoporosis.

## Conclusions

This is a report of a young man with OCA and short stature who was coincidentally found to have severe osteoporosis. This case report while attempting to review the literature also emphasizes the importance of further research into the prevalence of osteoporosis and other clinical features accompanying certain types of OCA and whether they are part of a syndrome similar to that of OOCHS or just coincidental findings.
